# Hepatic safety of sintilimab versus pembrolizumab in advanced non-small cell lung cancer: a retrospective observational cohort study

**DOI:** 10.3389/fimmu.2026.1808972

**Published:** 2026-06-24

**Authors:** Pan Ma, Yumeng Zhou, Xin Zhang, Quanfeng Zhao, Yu Peng, Hailong Jiang, Linli Xie, Yan Liao

**Affiliations:** 1 Department of Pharmacy, The First Affiliated Hospital of Army Medical University, Chongqing, China; 2 Department of Disease Prevention and Control, Daping Hospital, State Key Laboratory of Trauma and Chemical Poisoning, Army Medical University, Chongqing, China; 3 Department of Oncology, The First Affiliated Hospital of Army Medical University, Chongqing, China

**Keywords:** hepatotoxicity, immune checkpoint inhibitor, non-small cell lung cancer, pembrolizumab, real-world, sintilimab

## Abstract

**Background:**

Sintilimab and Pembrolizumab are widely used in non-small cell lung cancer (NSCLC), yet direct comparisons of their safety profiles, particularly hepatotoxicity, are lacking. Given the differences in binding affinities and the complexity of patients in real-world settings, this study aims to provide a head-to-head comparison of their hepatic safety to guide clinical decision-making.

**Methods:**

We conducted a retrospective observational cohort study to evaluate potential differences in liver function damage between Sintilimab and Pembrolizumab, administered as monotherapy or combination with chemotherapy, in patients with advanced NSCLC. Patients treated with Sintilimab or Pembrolizumab at Southwest Hospital were included. Propensity score matching (PSM) was performed using height and treatment regimen as covariates to balance between-group differences. Cox proportional hazards models and Kaplan-Meier curves were used to assess differences in hepatotoxicity. Benjamini-Hochberg correction was applied for multiple comparisons across liver function indicators.

**Results:**

A total of 222 patients with advanced NSCLC treated with Sintilimab or Pembrolizumab were included. The combination chemotherapy regimens did not differ significantly between the two groups. The overall incidence of hepatic-related adverse events (AEs) was 17.1% in the Sintilimab group and 18.1% in the Pembrolizumab group. No significant differences were observed in AEs related to liver function indicators, including aspartate aminotransferase (AST), alanine aminotransferase (ALT), alkaline phosphatase (ALP), gamma-glutamyl transferase (GGT), and total bilirubin (TBIL). Kaplan-Meier analysis revealed similar risks of hepatotoxicity between the two treatment regimens. No Grade 3 or higher hepatotoxicity events were observed in either group.

**Conclusions:**

In this retrospective observational cohort of Chinese patients with advanced NSCLC, Sintilimab and Pembrolizumab demonstrated comparable hepatic safety profiles, with no significant difference in the incidence, timing, or severity of hepatotoxicity. These findings suggest that Sintilimab may represent a clinically acceptable PD-1 inhibitor option without evidence of increased hepatic risk compared with Pembrolizumab in this setting. Further prospective, multi-center studies with larger and multi-ethnic cohorts are warranted to validate these findings.

## 1 Introduction

In recent decades, immune checkpoint inhibitors (ICIs), particularly programmed cell death-1 (PD-1) inhibitors and programmed cell death-ligand 1 (PD-L1) inhibitors, have revolutionized the treatment landscape for non-small cell lung cancer (NSCLC) (1). However, the immune activation induced by these therapies can trigger immune-related adverse events (irAEs) due to the disruption of immune tolerance (2). Among these, ICI-induced liver injury is a common yet challenging adverse event. Its early clinical symptoms are often nonspecific, complicating timely diagnosis. Without prompt intervention, hepatotoxicity can progress to fatal liver failure, posing a severe threat to patient survival (3). Previous studies have demonstrated that the development of hepatotoxicity is associated with a poorer prognosis; specifically, both progression-free survival (PFS) (64 days vs. 122 days) and overall survival (OS) (184 days vs. 427 days) are significantly reduced in affected patients (4). Consequently, the surveillance and management of ICI-associated hepatotoxicity warrant heightened clinical attention.

Pembrolizumab, the first highly selective PD-1 inhibitor, has established itself as a standard first-line treatment for advanced NSCLC globally, including in China (5). It has demonstrated dramatic significant efficacy both as monotherapy and in combination with platinum-based chemotherapy (6, 7). Similarly, Sintilimab is a highly selective, fully human monoclonal anti-PD-1 antibody developed by a Chinese manufacturer. It has also been approved in China for use in combination with chemotherapy for untreated NSCLC patients (8, 9). While both agents are potent PD-1 inhibitors, they possess distinct molecular characteristics. Sintilimab is a fully human IgG4 monoclonal antibody with a unique binding epitope and a higher affinity for the PD-1 receptor compared to Pembrolizumab (10). Given that Sintilimab exhibits a higher binding affinity for PD-1 and engages a distinct epitope compared to Pembrolizumab, it is plausible that these molecular differences may result in differential degrees of immune activation in hepatic tissue, thereby leading to distinct hepatotoxicity profiles. Therefore, a direct comparison of the hepatic safety of these two agents is warranted.

Despite their widespread use, direct “head-to-head” comparisons between these two agents are lacking. Most existing data are derived from separate clinical trials with highly restrictive inclusion criteria, which often exclude patients with borderline liver function, comorbidities, or those requiring complex combination therapies. As a result, the “true” incidence and patterns of hepatotoxicity in routine clinical practice remain unclear. To date, only a few real-world studies have compared the efficacy and safety of PD-1 inhibitors in NSCLC (11, 12), and a randomized controlled trial in China reported generally similar safety profiles between Sintilimab and Pembrolizumab (13). However, no study has specifically investigated the differences in hepatotoxicity between these two drugs in a routine clinical practice setting involving a broader, more heterogeneous patient population.

Therefore, we conducted this retrospective observational cohort study to directly compare the hepatotoxicity profiles of Sintilimab and Pembrolizumab in patients with advanced NSCLC. By reviewing the medical records of 648 patients, we aimed to evaluate potential differences in liver-related irAEs and provide evidence-based guidance for clinical decision-making.

## 2 Materials and methods

### 2.1 Subjects

This retrospective study was conducted at the First Affiliated Hospital of Army Medical University from April 2019 to April 2023. Eligible patients were aged 18 years or older, diagnosed with stage IV advanced NSCLC, and had a confirmed pathological diagnosis of NSCLC, including squamous cell carcinoma, adenocarcinoma, and large cell carcinoma. Additionally, patients must have previously received PD-1 inhibitors (Sintilimab or Pembrolizumab) as monotherapy or in combination with chemotherapy for NSCLC and provided complete baseline information. Patients were excluded if they were pregnant, had incomplete medical records, or exhibited a high missing rate of medical test indicators. The safety observation period for each patient was capped at 12 treatment cycles (approximately 36 weeks at a standard 3-week cycle interval) to standardize the follow-up duration. This observation window was selected based on published evidence indicating that ICI-induced hepatotoxicity typically manifests within 3–9 weeks of treatment initiation, with the majority of events occurring within the first 6 months of therapy19. Thus, 12 cycles adequately capture both early-onset and delayed hepatotoxicity. Patients were not excluded if they discontinued treatment early due to AEs or disease progression. Their data were included up to the point of discontinuation, ensuring that early hepatotoxicity events were captured. Ultimately, 426 patients treated with Sintilimab and 222 patients treated with Pembrolizumab met the eligibility criteria and were included in the study (P = 0.002).

### 2.2 Ethics approval and consent to participate

This study was approved by the Hospital Ethics Committee of the First Affiliated Hospital of Army Medical University ([B]KY2024117), and performed in accordance with the Declaration of Helsinki. This study collected medical record data obtained during previous clinical diagnosis and treatment for retrospective analysis. It did not involve human biological samples, and all data can be accessed through the hospital’s Electronic Medical Record System (EMRS). Therefore, informed consent was exempted in the ethical approval documents.

### 2.3 Data collection

Demographic information, NSCLC stage, treatment regimen and medical records were collected. Basic demographic data included age, gender, BMI (body mass index), nationality, smoking status and drinking status. Drug use history and clinical examination indicators, such as blood routine test results, were also extracted. Baseline viral hepatitis (HBV/HCV) serological data were not uniformly documented across all patient records; therefore, hepatitis status could not be included as a variable in the analysis. Treatment regimens for eligible patients included PD-1 inhibitor monotherapy and combination therapy with chemotherapy, categorized as follows: 1) Monotherapy with Sintilimab or Pembrolizumab; 2) Sintilimab or Pembrolizumab combined with platinum-based chemotherapeutic drugs; 3) Sintilimab or Pembrolizumab combined with other types of chemotherapeutic drugs. To further analyze the effects of different treatment regimens, we classified combination therapies involving PD-1 inhibitors and chemotherapy into more detailed categories: 1) PD-1 inhibitor + platinum + pemetrexed; 2) PD-1 inhibitor + platinum + paclitaxel; 3) PD-1 inhibitor + platinum + gemcitabine; 4) PD-1 inhibitor + platinum + pemetrexed/paclitaxel/gemcitabine + targeted drugs; 5) PD-1 inhibitor + targeted drugs; 6) PD-1 inhibitor + single agent chemotherapy; 7) Others.

Hepatotoxicity was graded according to the National Cancer Institute Common Terminology Criteria for Adverse Events (CTCAE), version 5.0. The assessment was based on laboratory parameters including alanine aminotransferase (ALT), aspartate aminotransferase (AST), alkaline phosphatase (ALP), gamma-glutamyl transferase (GGT), and total bilirubin (TBIL). A hepatic adverse event was defined as any elevation in these parameters reaching Grade 1 or higher.

### 2.4 Statistical analyses

Basic demographic and disease information for included subjects are presented as mean ± standard error for continuous variables and percentages (%) for categorical variables. For all eligible patients, we performed 1:1 matching using a propensity score matching method to balance potential confounding factors, resulting in 222 patients each in the Sintilimab and Pembrolizumab groups. Propensity Score Matching (PSM) was performed using the nearest neighbor algorithm with a caliper width of 0.2. The covariates included in the PSM model were height and treatment regimen (agent_type1), as these were the two variables that demonstrated statistically significant between-group imbalances before matching (height: P = 0.002; treatment regimen: P = 0.002; **Supplementary Table 1**). Other baseline characteristics, including age, gender, BMI, smoking status, and drinking status, were already balanced between groups before matching (all P > 0.05) and were further adjusted in the multivariable Cox regression analysis. We acknowledge that baseline liver function tests, viral hepatitis status, liver metastases, and hepatotoxic co-medications were not available in our dataset and could not be included in the PSM model.

Differences in the timing and incidence of irAEs associated with hepatotoxicity in NSCLC patients treated with Sintilimab or Pembrolizumab were compared using Kaplan-Meier survival analysis. The primary safety endpoint was the incidence of hepatic AEs. For the time-to-event analysis (Kaplan-Meier curves), the “event” was defined as the first occurrence of hepatotoxicity during the treatment period. Treatment cycles were used as the time unit for Kaplan-Meier analysis because patients received immunotherapy at varying intervals (every 2–3 weeks); one cycle approximately equals 21 days for standard regimens. Patients who did not experience hepatic AEs were censored at the date of their last follow-up or treatment discontinuation. Additionally, we compared the incidence and timing of liver-related irAEs in advanced NSCLC patients treated with PD-1 inhibitors, either with or without platinum-based chemotherapy.

We constructed models using a two-stage Cox proportional hazards regression approach. In the first stage, we built initial Cox regression model adjusted for single factor, including the type of PD-1 inhibitor, age, gender, BMI, nationality, smoking status, drinking status, and the use of combination therapy. In the second stage, factors significantly associated with the incidence of irAEs were included in a fully adjusted model.

Our results were presented as hazard ratios (HR) with 95% confidence intervals. Chi-square test was used to compare category variables between groups, and t-test was used to compare continuous variables between groups. Given that multiple liver function indicators (ALT, AST, ALP, GGT, TBIL) were compared simultaneously, the Benjamini-Hochberg false discovery rate (FDR) correction was applied to adjust for multiple comparisons. All of our statistical analyses were conducted using R software 4.2.3, and *P* < 0.05 was statistically significant.

## 3 Results

### 3.1 Patients and treatment characteristics

First, we retrospectively reviewed the medical records of 648 patients with advanced NSCLC treated with PD-1 inhibitors (426 treated with Sintilimab and 222 treated with Pembrolizumab). The mean height of patients treated with Pembrolizumab was significantly higher than that of patients treated with Sintilimab (164cm vs 162cm, P = 0.002) (**Supplementary Table 1**). Regarding treatment regimens, 48.2% of patients in the Pembrolizumab group received PD-1 inhibitors alone, compared to 34.3% in the Sintilimab group. Detailed subgroup analysis of PD-1 inhibitor combinations with chemotherapy revealed significant differences between the Sintilimab and Pembrolizumab groups.

After 1:1propensity score matching to balance confounding factors between groups, 222 patients from each group were included in the analysis. Among the 444 matched patients, the age range was 17.0 to 82.0 years (median age: 59.0 years) in the Sintilimab group and 24.0 to 89.0 years (median age: 59.5 years) in the Pembrolizumab group. In the Sintilimab group, 189 patients (85.1%) were women, with a mean BMI of 23.3 (± 3.14), while in the Pembrolizumab group, 174 patients (78.4%) were women, with a mean BMI of 23.0 (± 3.06). No significant differences were observed between the Sintilimab and Pembrolizumab groups in terms of age (P = 0.537), smoking status (P = 0.114), drinking status (P = 0.445), use of combination chemotherapy (P = 0.342), or detailed chemotherapy regimens (P = 0.155) (**Table 1**).

**Table 1:** Baseline characteristics after 1:1 matching.

Characteristic	Sintilimab	Pembrolizumab	*P*-value
N	222	222	
Age			
Mean (SD)	58.9 (10.8)	59.5 (11.2)	0.537
Median [Min, Max]	59.0 [17.0, 82.0]	59.5 [24.0, 89.0]	
Gender			
Female	33 (14.9%)	48 (21.6%)	0.0849
Male	189 (85.1%)	174 (78.4%)	
Height			
Mean (SD)	164 (6.70)	164 (6.82)	0.681
Median [Min, Max]	165 [140, 180]	165 [142, 179]	
Weight			
Mean (SD)	62.8 (9.54)	62.0 (9.80)	0.379
Median [Min, Max]	62.3 [40.0, 85.0]	61.0 [41.0, 94.0]	
BMI			
Mean (SD)	23.3 (3.14)	23.0 (3.06)	0.419
Median [Min, Max]	23.2 [15.9, 32.5]	22.9 [16.2, 31.2]	
Smoke			
No	74 (33.3%)	91 (41.0%)	0.114
Yes	145 (65.3%)	128 (57.7%)	
Missing	3 (1.4%)	3 (1.4%)	
Drink			
No	118 (53.2%)	109 (49.1%)	0.445
Yes	102 (45.9%)	111 (50.0%)	
Missing	2 (0.9%)	2 (0.9%)	
Agent type1			
1	93 (41.9%)	107 (48.2%)	0.342
2	62 (27.9%)	50 (22.5%)	
3	67 (30.2%)	65 (29.3%)	
Agent_type2			
1	7 (3.2%)	5 (2.3%)	0.155
2	36 (16.2%)	38 (17.1%)	
4	22 (9.9%)	25 (11.3%)	
5	25 (11.3%)	11 (5.0%)	
6	93 (41.9%)	109 (49.1%)	
7	39 (17.6%)	34 (15.3%)	

Agent type1:.

1. Monotherapy with Sintilimab or Pembrolizumab;

2. Sintilimab or Pembrolizumab combined with platinum-based chemotherapeutic drugs;

3. Sintilimab or Pembrolizumab combined with other types of chemotherapeutic drugs.

Agent_type2:.

1. PD-1 inhibitor + platinum + pemetrexed;

2. PD-1 inhibitor + platinum + paclitaxel;

3. PD-1 inhibitor + platinum + gemcitabine;

4. PD-1 inhibitor + platinum + pemetrexed/paclitaxel/gemcitabine + targeted drugs;

5. PD-1 inhibitor + targeted drugs;

6. PD-1 inhibitor + single agent chemotherapy;

7. Others.

### 3.2 Characteristics of adverse events associated with hepatotoxicity

The hepatotoxicity characteristics of the included individuals are presented in **Table 2** and **Supplementary Table S2**. Before 1:1 matching (**Supplementary Table 2**), the incidences of AE associated with hepatotoxicity were similar between patients treated with Sintilimab and Pembrolizumab. The mean medication cycles for PD-1 monotherapy were 5.03 for Sintilimab and 8.19 for Pembrolizumab, while the mean cycles for PD-1 combination therapy were 22.9 for the Sintilimab group and 13.0 for the Pembrolizumab group. Different treatment regimens showed variations in the timing of irAEs occurrence. For Sintilimab, the mean cycle for developing irAEs was 4.59 for monotherapy and 10.1 for combination therapy, compared to 5.93 for Pembrolizumab monotherapy and 11.2 for combination therapy.

**Table 2:** Description of AEs and medication cycles after 1:1matching.

Variable	Sintilimab	Pembrolizumab	*P*-value
N	222	222	
AST_AE			
0	214 (96.4%)	207 (93.2%)	0.34
1	8 (3.6%)	15 (6.8%)	
ALT_AE			
0	209 (94.1%)	203 (91.4%)	0.45
1	13 (5.9%)	19 (8.6%)	
ALP_AE			
0	212 (95.5%)	215 (96.8%)	0.0555
1	9 (4.1%)	7 (3.2%)	
GGT_AE			
0	216 (97.3%)	220 (99.1%)	0.247
1	2 (0.9%)	0 (0%)	
TBIL_AE			
0	200 (90.1%)	206 (92.8%)	0.672
1	20 (9.0%)	16 (7.2%)	
AE			
0	184 (82.9%)	182 (82.0%)	0.961
1	38 (17.1%)	40 (18.1%)	
pd1_total_cycle (cycles)			
Mean (SD)	4.99 (3.72)	8.19 (7.15)	<0.001
Median [Min, Max]	4.00 [2.00, 26.0]	5.00 [2.00, 34.0]	
ca_total_cycle (cycles)			
Mean (SD)	22.1 (17.6)	13.0 (9.75)	<0.001
Median [Min, Max]	17.0 [4.00, 105]	10.0 [2.00, 53.0]	
AE_period1 (days)			
Mean (SD)	4.57 (2.88)	5.93 (3.92)	<0.001
Median [Min, Max]	4.00 [2.00, 12.0]	4.00 [2.00, 12.0]	
AE_period2 (days)			
Mean (SD)	10.4 (9.13)	11.2 (8.50)	0.352
Median [Min, Max]	7.00 [2.00, 54.0]	9.00 [1.00, 45.0]	

ALT, alanine aminotransferase; AST, aspartate aminotransferase; ALP, alkaline phosphatase; GGT, gamma-glutamyl transferase; TBIL, total bilirubin; AE, adverse event; pd1_total_cycle, total cycles of PD-1 inhibitor (Sintilimab or Pembrolizumab) treatment; ca_total_cycle, total cycles of combined chemotherapy treatment; AE_period1, time from the first dose of immunotherapy to the first occurrence of hepatotoxicity; AE_period2, duration from the onset of hepatotoxicity to recovery or stabilization. Definitions: 0 = no hepatotoxicity (Grade 0); 1 = occurrence of hepatotoxicity (Any Grade, from Grade 1 to 4, according to CTCAE v5.0).

After 1:1 matching (**Table 2**), the trend in medication cycles for irAE occurrence remained consistent. The mean cycle was 4.99 for Sintilimab monotherapy and 22.1 for combination therapy. The mean treatment duration for patients developing irAEs was 4.57 cycles for Sintilimab monotherapy and 10.4 cycles for Sintilimab combination therapy.

### 3.3 Characteristics of the medication cycle

**Figure 1:**
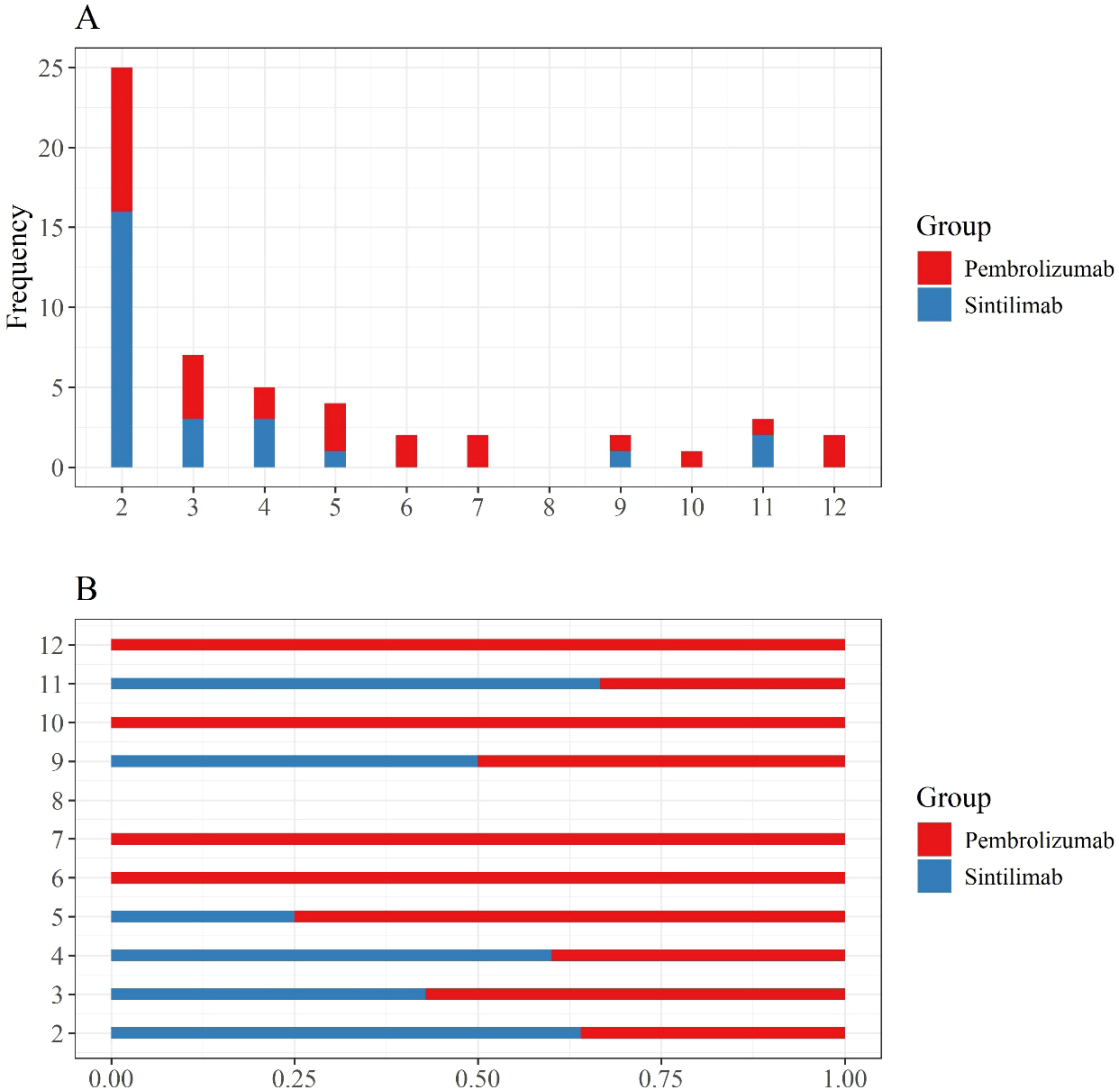
Distribution of the onset cycle of immune-related hepatotoxicity. **(A)** Frequency distribution of the first occurrence of hepatic adverse events (AEs) across treatment cycles for Sintilimab and Pembrolizumab groups. **(B)** Relative proportion (percentage) of hepatic AE onset per cycle. one cycle ≈ 21 days for standard regimens.

Among patients treated with Sintilimab, 38 developed AEs, including 26 (11.7%) with grade 1 hepatotoxicity and 12 (5.4%) with grade 2 hepatotoxicity (**Table 2**). No Grade 3 or Grade 4 hepatotoxicity events were observed in either group. The highest number of AEs occurred during the second medication cycle, with 16 AEs observed. Subsequently, the number of AEs gradually decreased as medication cycles progressed but re-emerged during the 11th and 12th cycles. Patients treated with Pembrolizumab exhibited a similar trend, although AEs occurred over more cycles (**Figure 1A**).

By comparing the treatment regimens of patients who experienced AEs in each medication cycle, we found that the proportion of AEs in Sintilimab-treated patients tended to decrease over time, while the proportion in Pembrolizumab-treated patients increased (**Figure 1B**).

### 3.4 Association between medicine and hepatic-related adverse events

**Figure 2:**
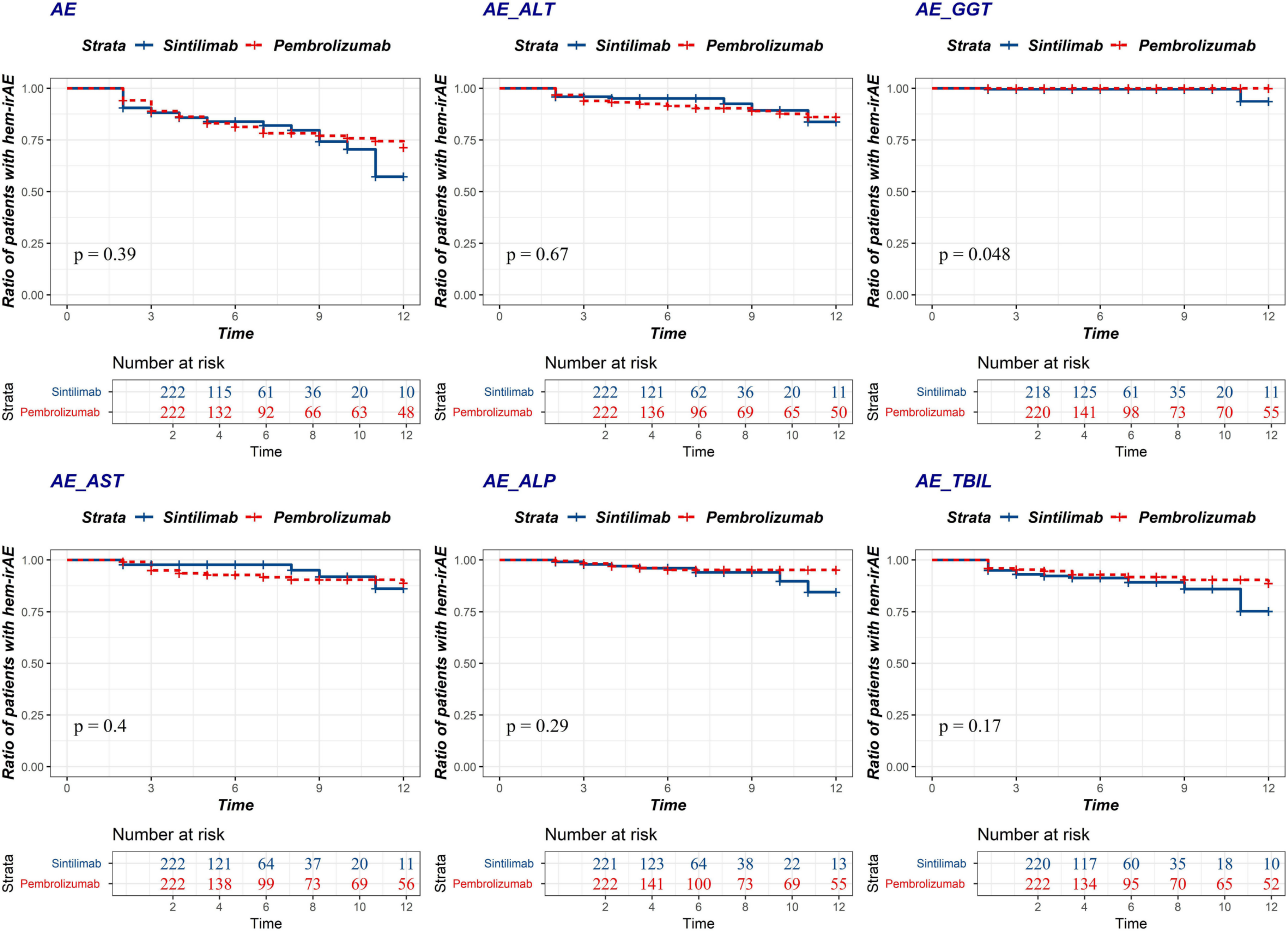
Cumulative incidence of hepatic adverse events suggestive of immune checkpoint inhibitor-associated hepatotoxicity. Kaplan-Meier curves represent the probability of remaining free from hepatic adverse events over treatment cycles. The analysis compared the Sintilimab group (blue line) and the Pembrolizumab group (red line) after 1:1 propensity score matching. The log-rank test was used to determine the statistical significance of the difference between the two groups. The “Risk Table” below indicates the number of patients at risk for hepatic AEs at each cycle interval. one cycle ≈ 21 days for standard regimens.

Using the Kaplan-Meier method and Cox proportional hazards model, we found no significant differences in the incidence of hepatic-related AEs between Sintilimab- and Pembrolizumab-treated patients (HR = 0.82, 95%CI: 0.52-1.29). Similarly, the likelihood of AEs related to liver function indicators, including ALT (HR = 1.17, 95%CI: 0.57-2.39), AST (HR = 1.45, 95%CI: 0.61-3.47), ALP (HR = 0.59, 95%CI: 0.22-1.61), and TBIL (HR = 0.63, 95%CI: 0.32-1.23), showed no significant differences. However, the likelihood of AEs related to GGT was higher in Sintilimab-treated patients compared to Pembrolizumab-treated patients. A numerically higher frequency of GGT-related AEs was observed in the Sintilimab group (2 events, 0.9%) compared to the Pembrolizumab group (0 events); however, after applying Benjamini-Hochberg correction for multiple comparisons across the five liver function indicators, the borderline significance was not maintained (unadjusted P = 0.048; FDR-adjusted P = 0.24). Given the extremely low event count, this finding should be interpreted with caution and may represent a chance observation (**Figure 2**).

## 4 Discussion

Since the approval of the first PD-1 inhibitor, an increasing number of PD-1 and PD-L1 inhibitors have been approved by the FDA and NMPA, offering broader options for treating advanced NSCLC (14–16). As ICI therapy gradually replaces chemotherapy as the standard of care, there is an immediate need to differentiate the toxicity profiles of specific inhibitors to optimize first-line treatment selection. This study was conducted to provide a direct, retrospective comparative analysis of hepatotoxicity between Sintilimab and Pembrolizumab in patients with advanced NSCLC.

A notable strength of this retrospective observational cohort study is its inclusion of a diverse patient population receiving various chemotherapy combinations, who are often excluded from randomized controlled trials (RCTs). To mitigate the potential bias arising from non-uniform treatment protocols, we utilized Propensity Score Matching (PSM) and incorporated the treatment regimen as a covariate. We found that the incidence of hepatotoxicity in patients treated with Sintilimab (17.1%) was not significantly different from those treated with Pembrolizumab (18.1%). This lack of statistical difference was further validated by our Cox proportional hazards model (HR = 0.82; 95% CI: 0.52-1.29; P = 0.386). Furthermore, individual liver function indicators—including AST, ALT, ALP, GGT, and TBIL-showed comparable risk profiles between the two groups (all P > 0.05). Notably, the borderline significance of GGT-related AEs (unadjusted P = 0.048) was not maintained after Benjamini-Hochberg correction for multiple comparisons (adjusted P = 0.24), and given the extremely low event count (2 vs. 0), this finding should be considered a potential chance observation rather than a true pharmacological difference. These findings suggest that Sintilimab and Pembrolizumab possess comparable hepatic safety, regardless of the treatment cycles or specific chemotherapy regimens employed.

The observed differences in treatment duration between the two groups warrant discussion. The longer combination therapy cycles for Sintilimab (22.1 vs. 13.0 cycles) reflect real-world prescribing patterns in China, where Sintilimab is often continued for extended maintenance cycles due to its lower cost and broader national medical insurance coverage. Conversely, the longer PD-1 monotherapy duration for Pembrolizumab (8.19 vs. 4.99 cycles) reflects its established use as a first-line monotherapy based on KEYNOTE trial evidence. Importantly, our Cox proportional hazards analysis uses time-to-event methodology, which inherently accounts for differential follow-up durations by treating patients as censored observations when they exit the study period.

An important observation in our study was that the frequency of hepatotoxicity peaked during the second medication cycle for both agents (**Figure 1A**). This pattern is neither a statistical anomaly nor a clinical drift; rather, it aligns with the pharmacological kinetics of immune-mediated liver injury. ICI-induced T-cell activation typically requires a priming phase, with hepatotoxicity often manifesting between 3 and 9 weeks after the initiation of therapy (17). Our findings reinforce the clinical necessity of intensive liver function monitoring during the first two months of treatment.

Although the precise mechanisms underlying differential hepatotoxicity between specific PD-1 inhibitors remain to be elucidated, the general pathophysiology of ICI-induced liver injury is thought to involve several pathways. Hepatocyte injury may be driven by overactivation of CD8+ T-cells, disruption of immune tolerance, reduction in regulatory T-cell function (18), epitope spreading from tumor antigens to self-antigens (19), and increased pro-inflammatory cytokine signaling, including tumor necrosis factor and interleukin-2 (20, 21). However, these mechanisms describe general immune checkpoint inhibitor class effects, and our study did not include immunophenotyping, cytokine profiling, liver biopsy, or biomarker analyses to determine drug-specific mechanistic differences between Sintilimab and Pembrolizumab.

The comparable safety profile of Sintilimab in our study is particularly noteworthy. Previous meta-analyses have suggested that Sintilimab-based combinations may even have lower withdrawal rates due to AEs compared to Pembrolizumab-based regimens (22). One potential hypothesis is that Sintilimab’s molecular structure as a fully human monoclonal antibody, which potentially lowers immunogenicity (23). Furthermore, Sintilimab’s higher binding affinity to the PD-1 receptor and its unique binding epitope might result in more targeted immune modulation and less off-target binding (10, 24). However, these hypotheses require validation through dedicated mechanistic studies.

To our knowledge, this is the first head-to-head retrospective comparative analysis of hepatotoxicity between Sintilimab and Pembrolizumab in NSCLC. However, several limitations must be acknowledged. First, the retrospective, single-center design may introduce inherent selection bias. Although we clarified that the “12-cycle” threshold was an observation window rather than a mandatory inclusion criterion, early treatment discontinuation remains a factor in real-world data. Additionally, late-onset hepatotoxicity occurring beyond 12 cycles may have been missed. Second, while PSM was employed, unmeasured confounders such as baseline hepatitis status or specific co-medications (e.g., hepatotoxic antibiotics or analgesics) might influence results. In particular, the absence of baseline viral hepatitis (HBV/HCV) serological data represents a significant limitation, especially given the high HBV carrier rate in China (approximately 6-8% of the general population). Both treatment groups likely included patients with chronic HBV infection, and the lack of stratification by hepatitis status may have influenced the observed hepatotoxicity rates. Third, since Sintilimab is primarily utilized in China, our findings require validation in global, multi-ethnic cohorts to confirm international applicability. Specifically, pharmacogenomic differences, including variations in HLA polymorphisms and drug metabolism enzymes across ethnic groups, may influence susceptibility to ICI-induced hepatotoxicity. The high baseline prevalence of chronic liver disease in the Chinese population further limits the generalizability of our findings to Western populations. Finally, the sample size in certain subgroups may limit the statistical power to detect subtle differences in high-grade (Grade ≥ 3) adverse events. Indeed, no Grade 3–4 hepatotoxicity events were observed in either group, suggesting that the study was underpowered to evaluate severe hepatotoxicity.

## 5 Conclusion

In this retrospective observational cohort study, no significant statistical differences in hepatotoxicity were detected between Sintilimab and Pembrolizumab, whether administered as monotherapy or in combination with chemotherapy, for the treatment of advanced NSCLC. These findings are primarily applicable to Chinese NSCLC populations. Given that Sintilimab does not demonstrate a higher hepatic risk compared to the international standard PD-1 inhibitor, it represents a safe and valuable immunotherapeutic option, particularly for patients at higher risk of liver dysfunction. Future research should focus on prospective, multi-center trials with larger cohorts incorporating baseline viral hepatitis screening and stratification to validate these findings and explore the long-term safety profiles of various PD-1 inhibitors. Additionally, investigating the molecular biomarkers that predict individual susceptibility to immune-related hepatotoxicity through immunophenotyping, cytokine profiling, and pharmacogenomic analyses will be a crucial next step to enhance clinical applicability and achieve personalized immunotherapy.

## Data Availability

The original contributions presented in the study are included in the article/**Supplementary Material**, further inquiries can be directed to the corresponding author/s.

## Supplementary material

Supplementary Table 1 Baseline characteristics before 1:1 matching. Agent type1: 1. Monotherapy with Sintilimab or Pembrolizumab; 2. Sintilimab or Pembrolizumab combined with platinum-based chemotherapeutic drugs; 3. Sintilimab or Pembrolizumab combined with other types of chemotherapeutic drugs. Agent_type2: 1. PD-1 inhibitor + platinum + pemetrexed; 2. PD-1 inhibitor + platinum + paclitaxel; 3. PD-1 inhibitor + platinum + gemcitabine; 4. PD-1 inhibitor + platinum + pemetrexed/paclitaxel/gemcitabine + targeted drugs; 5. PD-1 inhibitor + targeted drugs; 6. PD-1 inhibitor + single agent chemotherapy; 7. Others.

Supplementary Table 2 Description of AEs and medication cycles before 1:1matching. pd1_total_cycle: the total number of cycles of use of PD-1 drugs; ca_total_cycle:the total number of cycles of use of anticancer drugs; AE_period1:number of cycles of pd1 drug use before the occurrence of AEs; AE_period2:number of cycles of anticancer drug use before the occurrence of AEs.
