# Supplementary material for: Hepatic safety of sintilimab versus pembrolizumab in advanced non-small cell lung cancer: a retrospective observational cohort study
Source: Front Immunol. 2026 Jun 24;17:1808972. doi: 10.3389/fimmu.2026.1808972 (PMC13341631; doi:10.3389/fimmu.2026.1808972)
Supplement: Supplementary Table 1 — Baseline characteristics before 1:1 matching. Agent type1: 1. Monotherapy with Sintilimab or Pembrolizumab; 2. Sintilimab or Pembrolizumab combined with platinum-based chemotherapeutic drugs; 3. Sintilimab or Pembrolizumab combined with other types of chemotherapeutic drugs. Agent_type2: 1. PD-1 inhibitor + platinum + pemetrexed; 2. PD-1 inhibitor + platinum + paclitaxel; 3. PD-1 inhibitor + platinum + gemcitabine; 4. PD-1 inhibitor + platinum + pemetrexed/paclitaxel/gemcitabine + targeted drugs; 5. PD-1 inhibitor + targeted drugs; 6. PD-1 inhibitor + single agent chemotherapy; 7. Others. [file Table1.docx]

**Table S1.** Baseline characteristics before 1:1 matching.

|  | **Sintilimab** | **Perbrolizumab** | ***P*-value** |
| --- | --- | --- | --- |
| **N** | 426 | 222 |  |
| **Age** |  |  |  |
| Mean (SD) | 59.0 (10.1) | 59.5 (11.2) | 0.553 |
| Median [Min, Max] | 58.0 [17.0, 82.0] | 59.5 [24.0, 89.0] |  |
| **Gender** |  |  |  |
| Female | 107 (25.1%) | 48 (21.6%) | 0.334 |
| Male | 319 (74.9%) | 174 (78.4%) |  |
| **Height** |  |  |  |
| Mean (SD) | 162 (7.57) | 164 (6.82) | 0.002 |
| Median [Min, Max] | 163 [140, 185] | 165 [142, 179] |  |
| **Weight** |  |  |  |
| Mean (SD) | 61.4 (9.76) | 62.0 (9.80) | 0.480 |
| Median [Min, Max] | 60.2 [37.0, 87.0] | 61.0 [41.0, 94.0] |  |
| **BMI** |  |  |  |
| Mean (SD) | 23.3 (3.03) | 23.0 (3.06) | 0.242 |
| Median [Min, Max] | 23.3 [15.9, 32.5] | 22.9 [16.2, 31.2] |  |
| **Smoke** |  |  |  |
| No | 175 (41.1%) | 91 (41.0%) | 1.000 |
| Yes | 244 (57.3%) | 128 (57.7%) |  |
| Missing | 7 (1.6%) | 3 (1.4%) |  |
| **Drink** |  |  |  |
| 0 | 248 (58.2%) | 109 (49.1%) | 0.030 |
| 1 | 174 (40.8%) | 111 (50.0%) |  |
| Missing | 4 (0.9%) | 2 (0.9%) |  |
| **agent_type1** |  |  |  |
| 1 | 146 (34.3%) | 107 (48.2%) | 0.002 |
| 2 | 151 (35.4%) | 50 (22.5%) |  |
| 3 | 129 (30.3%) | 65 (29.3%) |  |
| **agent_type2** |  |  |  |
| 1 | 19 (4.5%) | 5 (2.3%) | 0.002 |
| 2 | 88 (20.7%) | 38 (17.1%) |  |
| 4 | 43 (10.1%) | 25 (11.3%) |  |
| 5 | 49 (11.5%) | 11 (5.0%) |  |
| 6 | 147 (34.5%) | 109 (49.1%) |  |
| 7 | 80 (18.8%) | 34 (15.3%) |  |

**Table S2.** Description of AEs and medication cycles before 1:1matching.

|  | **Sintilimab** | **Perbrolizumab** | ***P*-value** |
| --- | --- | --- | --- |
| **N** | 426 | 222 |  |
| **AST_AE** |  |  |  |
| 0 | 410 (96.2%) | 207 (93.2%) | 0.214 |
| 1 | 16 (3.8%) | 15 (6.8%) |  |
| **ALT_AE** |  |  |  |
| 0 | 402 (94.4%) | 203 (91.4%) | 0.161 |
| 1 | 24 (5.6%) | 19 (8.6%) |  |
| **ALP_AE** |  |  |  |
| 0 | 408 (95.8%) | 215 (96.8%) | 0.195 |
| 1 | 17 (4.0%) | 7 (3.2%) |  |
| **GGT_AE** |  |  |  |
| 0 | 419 (98.4%) | 220 (99.1%) | 0.555 |
| 1 | 3 (0.7%) | 0 (0%) |  |
| **TBIL_AE** |  |  |  |
| 0 | 395 (92.7%) | 206 (92.8%) | 0.790 |
| 1 | 29 (6.8%) | 16 (7.2%) |  |
| **AE** |  |  |  |
| 0 | 361 (84.7%) | 182 (82.0%) | 0.534 |
| 1 | 65 (15.2%) | 40 (18.1%) |  |
| **pd1_total_cycle** |  |  |  |
| Mean (SD) | 5.03 (3.54) | 8.19 (7.15) | <0.001 |
| Median [Min, Max] | 4.00 [2.00, 26.0] | 5.00 [2.00, 34.0] |  |
| **ca_total_cycle** |  |  |  |
| Mean (SD) | 22.9 (16.6) | 13.0 (9.75) | <0.001 |
| Median [Min, Max] | 18.5 [3.00, 105] | 10.0 [2.00, 53.0] |  |
| **AE_period1** |  |  |  |
| Mean (SD) | 4.59 (2.82) | 5.93 (3.92) | <0.001 |
| Median [Min, Max] | 4.00 [2.00, 12.0] | 4.00 [2.00, 12.0] |  |
| **AE_period2** |  |  |  |
| Mean (SD) | 10.1 (8.36) | 11.2 (8.50) | 0.132 |
| Median [Min, Max] | 7.00 [2.00, 54.0] | 9.00 [1.00, 45.0] |  |

**pd1_total_cycle: the total number of cycles of use of PD-1 drugs;**

**ca_total_cycle：the total number of cycles of use of anticancer drugs;**

**AE_period1：number of cycles of pd1 drug use before the occurrence of AEs**

**AE_period2：number of cycles of anticancer drug use before the occurrence of Aes**
